# On the use of virtual simulation in radiotherapy of the intact breast

**DOI:** 10.1120/jacmp.v1i2.2646

**Published:** 2000-03-01

**Authors:** Wolfgang A. Tomé, Richard A. Steeves, Bhudatt P. Paliwal

**Affiliations:** ^1^ Departments of Human Oncology and Medical Physics Medical School University of Wisconsin Madison Wisconsin 53792; ^2^ Department of Human Oncology Medical School University of Wisconsin Madison Wisconsin 53792; ^3^ Departments of Human Oncology and Medical Physics Medical School University of Wisconsin Madison Wisconsin 53792

## Abstract

In this paper a method of breast cancer treatment planning using virtual simulation implemented at the Department of Human Oncology at the University of Wisconsin is described. All patients in this procedure are placed in a custom vacuum mold in treatment position with both arms up to avoid collision with the CT scanner aperture. For all patients a CT scan of 5‐mm‐slice thickness is acquired. The ipsilateral and contralateral breast, the ipsilateral lung and the heart are delineated and a three‐dimensional plan is generated that tries to minimize the dose to the ipsilateral lung and heart while ensuring adequate coverage of the affected breast. Digitally reconstructed radiographs are used to verify the patient setup on the treatment machine. © *2000 American College of Medical Physics.*

PACS number(s): 87.53.–j

## I. INTRODUCTION

In 1997, approximately 181,600 new cases of breast cancer were diagnosed in the United States. There were only 43,900 deaths that year, indicating that the majority of patients with breast cancer are treated successfully with long‐term survival. This high survival rate may be attributed in part to annual mammography as a screening tool for middle‐aged and elderly women. This has led to the more frequent diagnosis of breast cancer at earlier stages, including breast cancer *in situ.* Therefore, it has become feasible to utilize breast‐conserving treatment for most breast cancer patients. In 1992, the American College of Surgeons, the American College of Radiology, and the American Cancer Society agreed that it would be appropriate to apply breast‐conserving therapy for most breast cancer patients who had tumors less than 5 cm in diameter and affected breasts large enough to allow lumpectomy with a good cosmetic result. The only contraindications for breast‐conserving therapy were prior radiotherapy to the same breast, pregnancy, collagen vascular disease, or a high probability of gross multicentric breast cancer or extensive *in situ* carcinoma.

The goals of breast irradiation are to eradicate microscopic foci of multicentric cancer that might be present after lumpectomy, by using moderate doses of radiation. Theoretically, 50 Gy to the breast should minimize the probability of local recurrence at the primary site from approximately 20% (in unirradiated patients) to 5–7% over a five‐year observation period. Breast conservation is associated with better self‐image (i.e., quality of life), but should be delivered so that the risk of complications is low and the cosmetic outcome should be acceptable (cf. Refs. 1 and 2)

In this paper we describe a technique for virtual simulation and three‐dimensional (3D) treatment planning, that employs the PINNACLE^3^ 3D Treatment Planning System (ADAC Laboratories, Milpitas, CA), used in our clinic for breast‐conserving radiotherapy. Virtual simulation may not be needed for breast radiotherapy since it is by its nature regional therapy, and is therefore not target driven (cf. Ref. 3). However, even though the whole breast is the target, virtual simulation enables the planner to avoid critical organs such as the lung and heart. Hence, the value of virtual simulation in breast therapy does not lie in the ability to conform as best as possible to a target, but as best as possible to avoid critical structures while ensuring adequate coverage of the breast.

Image segmentation allows the planner to take critical anatomical structures explicitly into account through volume rendering in the 3D beam's‐eye‐view display, and therefore shape the blocks such that the critical organs are avoided as far as possible, while ensuring adequate coverage of the breast. This is in contrast to conventional simulation, where the blocks are shaped by either following the chest wall as imaged on the simulation film or using a rotatable half beam block to approximate the chest wall (see, for example, Refs. 4–7). Lind *et al.* ^8^ have concluded that there is a clinically important reduction in pulmonary function in a subset of patients following locoregional radiotherapy for breast cancer and point out that attention should be paid to individual lung dose‐volume histograms. Furthermore, Das *et al.* ^9^ have concluded in a retrospective study of 108 patients receiving breast radiotherapy that virtual simulation provides accurate information of the percent of irradiated volume of lung and heart, and point out that this information, together with dose‐volume histograms, is essential in reducing pulmonary and cardiac complications. In addition, the planner can easily determine if the lateral beam will diverge into the contralateral breast, and can choose the gantry angle accordingly. Last, but not least, it should be pointed out that virtual simulation allows this to be done without the physical presence of the patient, hence sparing the patient a sometimes lengthy and uncomfortable simulation procedure.

In the next section we describe a technique of virtual simulation as implemented at the University of Wisconsin. For ease of setup and to avoid match‐line issues we treat our intact breast using a mono‐isocentric technique (see, for example, Refs. 10–12). In the technique we employ the breast lies in the inferior half of the treatment field and a supraclavicular field can be easily matched in the superior half of the treatment field if therapeutically indicated.

## II. CT PROTOCOL FOR 3D PLANNING

When the patient arrives at the CT scanner she is placed supine in a halfbody Vac‐Lok bag (Med‐Tec, Orange City, IO) on the scanner table. The patient is then positioned with both arms up and the scanner couch is driven through the scanner aperture to determine if the patient will collide with the CT scanner aperture. If the patient does not clear the scanner aperture we adjust the arm position so that the patient will clear the scanner aperture. If the arm position has been adjusted as much as possible and the patient still does not clear the scanner aperture, then the patient is scheduled for a regular simulation. If the patient clears the scanner aperture without problems we proceed with making the custom mold.

**Figure 1:**
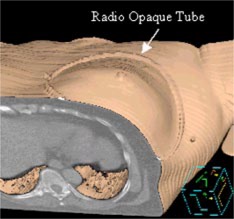
(Color) Three‐dimensional reconstruction of patient illustrating the placement of the radio‐opaque tube.

We pay particular attention to the reproducibility of shoulder and arm positions and that they are comfortable for the patient. Hence, we make sure that both arms and the shoulders are well outlined in the mold. We found that reproducibility of arm and shoulder position is one of the most important factors that determines the day‐to‐day reproducibility of the setup on the treatment machine. For this reason we have moved away from the use of a breast board, which did not provide a reproducible patient position on a day‐to‐day basis. The affected breast then is palpated and the breast parenchyma is outlined with a radio‐opaque tube having an outer diameter of 2.7 mm and an inner diameter of 1.5 mm (Cook, Bloomington, IN). Three fiducial markers are placed in anatomically stable regions. We have chosen these points to be the anterior midline over the sternum, a point on the skin near the ipsilateral lower axilla, and a point on the skin near the contralateral lower axilla. Of these three points the anterior midline sternum point is the anatomically most stable, and we make all our shifts from this point. It is important to realize that these fiducial points are the only link between the real and virtual patient. It should be emphasized, however, that this link is only as good as the reproducibility of the positioning of the patient with respect to the custom mold (see comments above) and the positioning of the vacuum mold with respect to the patient support system (treatment machine couch). If either of these fail to be reproducible, then the link between the real patient and virtual model is broken. Hence, particular attention is paid to these aspects. For this reason the points are marked with lead BBs during the CT scan and are tattooed afterwards. Once the fiducial points are marked the upper and lower limits of CT scans are set. The upper limit is chosen such that the first CT slice starts just below the mandible and the lower limit is chosen such that the entire breast and the BBs marking the points on the skin near the ipsilateral and contralateral lower axilla are included in the scan volume. CT images are acquired with 5 mm spacing. Figure 1 shows a surface rendering of a patient on the treatment planning system. One can clearly observe the radio‐opaque tube, which outlines the extent of the breast parenchyma as determined by the physician's palpation.

## III. VIRTUAL SIMULATION/PLANNING

### A. Registration of the patient in the treatment planning system

**Figure 2:**
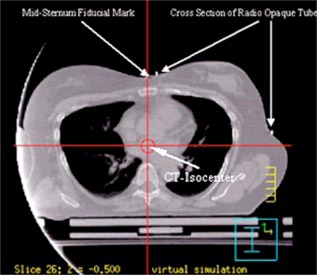
(Color) The CT‐isocenter is placed at the intersection of the perpendicular through the anterior midsternum BB and the coronal plane passing through the BBs placed on the skin in the region of the lower axilla.

After the CT scans have been acquired they are transferred over the network to the treatment planning system and a treatment plan is generated. First, we place a point of interest (POI) called the “CT Isocenter” at the intersection of the perpendicular through the anterior midsternum BB and the coronal plane passing through the BBs placed on the skin in the region of the lower axilla as is illustrated in Fig. 2. Once the CT Isocenter has been identified, the laser localization point is set to the CT Isocenter so that the correct shifts (that yield the patient's beam isocenter) are obtained at the end of treatment planning.

The laser localization tool in the treatment planning system allows the user to select a POI with respect to which all other POIs are referenced; i.e., the user chooses the origin to which everything else is referenced. It is, therefore natural to choose the CT Isocenter since everything has to be referenced with respect to this point when one translates the treatment plan into reality. In fact, the patient has now been registered in the treatment planning system and the link between the virtual and real patient has been established.

The PINNACLE^3^ treatment planning system employs the following coordinate system. The origin of an image set lies in the lower left‐hand corner of the first transverse CT slice in an image set. The positive *x* axis points from right to left, the positive *y* axis points from the posterior to the anterior, and the positive *z* axis points from the superior to the inferior direction.

**Figure 3:**
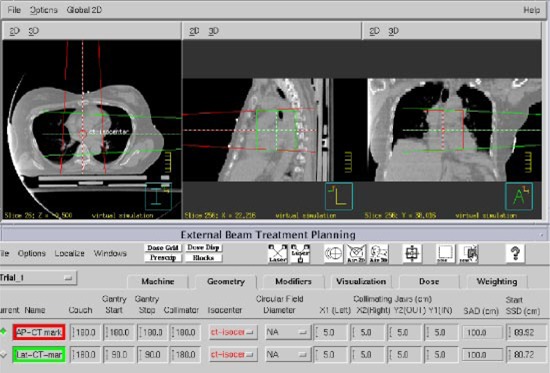
(Color) Placement of anterior and left lateral setup beams having the ‘CT Isocenter’ as their isocenter for a left breast. The anterior setup beam has an SSD=89.9 cm cm and the left lateral setup beam has an SSD=80.7 cm.

We take care that the *z* coordinate of the CT Isocenter coincides with the *z* coordinate of the CT slice that contains the midsternum fiducial mark. Next, an anterior beam and a right or left lateral beam (depending on which breast is treated) is added having the CT Isocenter as their isocenter. This is done to determine the AP and lateral setup SSDs (Fig. 3).

**Figure 4:**
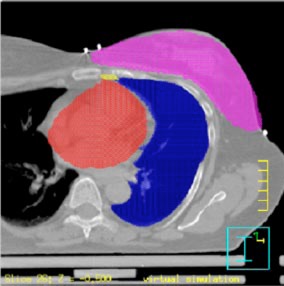
(Color) Typical image segmentation generated during the process of virtual simulation. The breast parenchyma, ipsilateral lung, heart, and (IM) nodes are shown.

This information is used at the treatment start to double‐check the initial setup to the CT Isocenter before the shifts to the beam isocenter are made. This is done since the tattoos placed on the patient at the time of CT do not lie in the same transverse plane. For convenience we then add another set of anterior and lateral beams. These beams are later set to the beam isocenter to

determine the AP and lateral setup SSDs for the beam isocenter, once it has been determined (see below) and the treatment plan has been generated. After this initial phase, we outline the ipsilateral and contralateral breast parenchyma [as well as the ipsilateral lung, heart, and internal mammary (IM) nodes] and define the superior and inferior borders by indicating the most superior and most inferior CT slice to be irradiated. Figure 4 shows the typical image segmentation obtained in this process. We use the radio‐opaque tube as a visual guide to relate the impression of the breast exam with the anatomical information contained in each of the transverse slices. It should be pointed out that there is no special reason other than convenience of illustration to follow the sequence of events outlined above. One could just as well start with outlining the anatomical structures and then proceed with the patient registration.

### B. Determining the isocenter and generating the treatment plan

**Figure 5:**
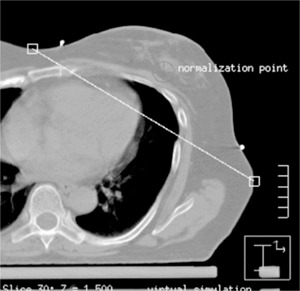
Placement of line that represents the intended posterior (deep) filed edge.

**Figure 6:**
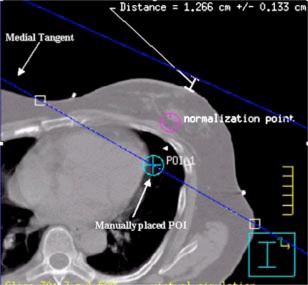
(Color) Shows an example of inadequate filed flash above the breast. The anterior field flash is less than 2 cm.

**Figure 7:**
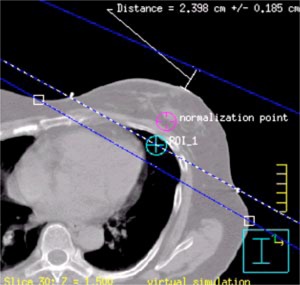
(Color) Shows an example of adequate anterior field flash after adjustments have been made. The anterior field flash is larger than 2 cm.

**Figure 8:**
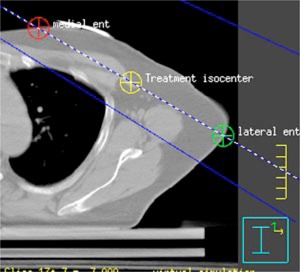
(Color) Treatment Isocenter is chosen as the midpoint of the line connecting the lateral and medial entrance points.

The next step is to determine the beam isocenter. Using the software utility “Auto place POI” in the treatment planning system, we place a POI in the center of the breast parenchyma. Next, on the CT slice closest to the *z* coordinate of this POI, the physician draws a line that represents the intended posterior (deep) field edge (Fig. 5). We use this line as a starting point for the selection of gantry angle. We then manually place a POI at the center of this line. Next we add the medial tangent beam and choose this POI as its provisionary isocenter. We then determine the gantry angle such that the central axis coincides with the drawn line. At this point we make sure that the medial tangent does not pass through the contralateral breast, that there is at least 2 cm of flash above the breast parenchyma, and that no more than 2–3 cm of lung are included in the treatment field by scanning through the CT slices above and below. If we find that the medial tangent passes through the contralateral breast, then the lateral point of the drawn line is moved posterior until the medial tangent does no longer pass through the contralateral breast. In case there is inadequate flash (Fig. 6), we move the manually placed POI anterior. To ensure adequate coverage of the breast the posterior jaw of the medial tangent beam is then opened by the amount the manually placed POI is moved anterior, and the gantry angle is changed so that the beam edge coincides with the line drawn (Fig. 7). We now set the true beam isocenter such that the breast will lie in the inferior half of the treatment field. On the most superior CT slice that defines the upper border of the treatment fields, the entrance and exit points of the central axis (CAX) of the medial tangent beam are marked by adding two points of interest. Note that the exit point of the medial tangent beam CAX is the entrance point of the lateral tangent beam CAX. The beam isocenter is then chosen as the midpoint of the line connecting the lateral and medial entrance points (Fig. 8).

This procedure ensures that the line drawn by the physician is the posterior field edge for the tangent fields. However, when the field size necessary to cover the breast exceeds 20 cm the above procedure is applied to the CT slice containing the manually placed POI. The *x* and *y* coordinates of the POI are modified such that the POI becomes the midpoint of the line connecting the entrance and exit points of CAX of medial tangent beam. The modified POI then becomes our beam isocenter and we follow the Siddon technique.^4^,^7^

**Figure 9:**
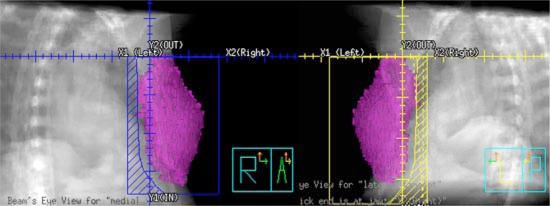
(Color) Beams‐eye‐view of the blocks for the lateral and medical tangent fields, which follows the outlined breast parenchyma.

The medial tangent beam is then copied and opposed to create the lateral tangent beam. If the isocenter has been moved anteriorly, then the gantry angle is changed so that the lateral tangent beam edge aligns with the line drawn by the physician. A 45° wedge is added to the lateral tangent beam, and, if needed, a 15° to 30° wedge may later be added to the medial tangent beam to improve dose homogeneity.^13^ The physician draws the blocks for both the medial and lateral tangent fields so that the treatment portals follow the outlined breast parenchyma (Fig. 9).

**Figure 10:**
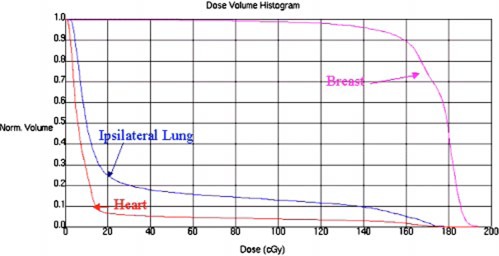
(Color) Dose volume histogram for an acceptable treatment plan. Cumulative dose volume Histograms for the breast parenchyma, lung, and heart are shown.

If the mono‐isocentric technique is used, the POI placed in the center of the breast parenchyma becomes the dose prescription point. We normalize the dose to this point, since it lies approximately in the center of the treatment field. This minimizes the dose inhomogeneity across the treatment volume. Doses are calculated using heterogeneity corrections and the beams are

weighted so that the prescription isodose line encircles most of the drawn‐in breast parenchyma (see discussion below). Figure 10 shows a typical dose‐volume histogram (DVH) for an acceptable treatment plan.

As can be seen from Fig. 10 it seems that only 50% of the target volume receives the prescribed dose of 1.8 Gy per fraction. As can be seen from Fig. 4 the breast extends all the way to the skin surface. Hence, some of the target volume lies in the build region, and will therefore receive a dose less than the prescribed dose. That this effect is quite significant can be seen from the following example. Approximate the breast by a semicircle of a radius of 6 cm, then a 1.0‐cm‐thick rim between 5 and 6 cm corresponds to 42% of the total volume. Therefore, it is not surprising that we have only 50% coverage of the target volume.

**Figure 11:**
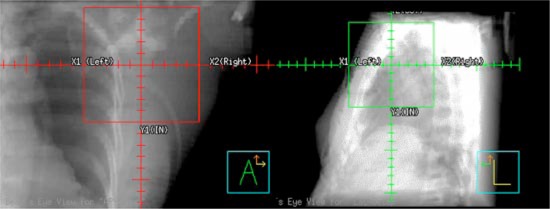
(Color) AP and left lateral digitally reconstructed radiographs for setup verification of the treatment isocenter (true beam isocenter).

**Figure 12:**
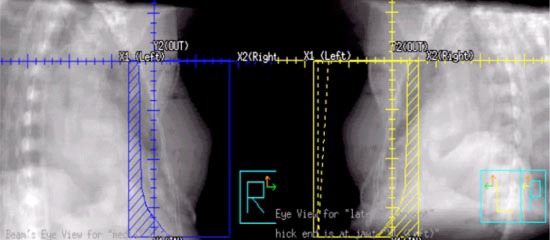
(Color) Lateral and medical tangent digitally reconstructed radiographs for setup verification of the lateral and medial tangent treatment field. The heel of lthe wedge is towards the $X_{1}$ (left) yaw as indicated in the DDR of the lateral tangent field on the right.

Once the treatment plan is deemed acceptable, we generate digitally reconstructed radiographs (DRRs) for AP and lateral setup beams to verify the beam isocenter as well as tangential treatment fields. We make sure that if there is a wedge in the beam, the wedged beam is printed so that the wedge direction can be verified (Figs. 11 and 12).

### C. Initial treatment setup

On the first day of treatment the patient is placed supine in the custom‐made mold that was manufactured at the time of the CT scan. The patient is then realigned to the CT marks and the AP and lateral SSDs are verified. We use the shifts that were determined at the time of virtual simulation to move the patient to the intended beam isocenter, and the AP and lateral SSDs for the beam isocenter are verified. Once it has been determined that the SSDs are correct, AP and lateral port films are obtained which are then compared to the AP and lateral isocenter DDRs, respectively. If the physician deems the setup satisfactory, we set the medial tangent field and a port film is obtained, and compared to the medial tangent field DRR. Once approved, the medial tangent field is treated, and one proceeds to the lateral tangent field for which we again obtain a port film. After the physician has approved the lateral tangent field port film, the wedge is inserted, the wedge direction is visually verified by the DDR indicating the wedge direction, and treatment is initiated. It typically takes between 25 and 35 min to execute a new start.

## IV. DISCUSSION

Besides making conventional simulation unnecessary, the application of 3D treatment planning allows us to be more precise about including within the treatment beams all tissues at risk for cancer recurrence. It also allows one to define treatment fields based on segmented images, i.e., based on a CT anatomy of the breast, lung, and heart. Since the planner can see each of these structures, treatment fields can be designed that balance the need for adequate coverage of the target volume with conformal avoidance of the lung and heart. This latter benefit is one of the major advantages of virtual simulation over conventional simulation. The radiation oncology team (physician, physicist, and dosimetrist) can generate a treatment plan based on quantitative data (cf. Ref. 9) instead of relying on radiological/anatomical landmarks. It is hoped that this will lead to fewer side effects (cf. Refs. 8 and 9) at equal tumor control rates. In addition, virtual simulation also opens the door to advanced treatment techniques such as the use of custom compensators (cf. Ref. 14) and IMRT (cf. Ref. 15).

## DEDICATION

This paper is dedicated to the memory of Judith Stitt, M.D., who has been an advocate for women's health issues and has been an avid supporter in the implementation of virtual simulation for treatment of breast cancer in our clinic.
